# The Otto Warburg International Summer School and Workshop on Networks and Regulation

**DOI:** 10.1186/1471-2105-8-S6-S1

**Published:** 2007-09-27

**Authors:** Peter F Arndt, Martin Vingron

**Affiliations:** 1 Max Planck Institute for Molecular Genetics, Ihnestr. 73, 14195 Berlin, Germany

## Introduction

The Otto Warburg International Summer School and Workshop 2005, held in Berlin, Germany, focussed on the topic of "Networks and Regulation", which is a very active field of research these days. The lecturers at the school were asked to give tutorial introductions that would allow the students to follow a research talk presented towards the end of the workshop. Overall, these lectures presented material starting where the text books stop and bridged to the current research front. The lectures were very well received by the students, prompting the lecturers to jointly work on a volume introducing the current research in this area.

The papers presented here are more or less based on the oral lectures, with some of the papers sticking very closely to the material presented then, and others going into more depth on particular questions. Not all presentations are represented and, vice versa, one paper has been included now which did not arise from a lecture. We felt it was simply missing in the context of the summer school and the current volume gave us the chance to fill the gap. The web site of the summer school () holds powerpoint presentations and video streams for most of the lectures.

## Presented material

The first paper by Silke Sperling [1] gives an introduction to transcriptional regulation as the basic theme of the summer school. Many aspects of the transcription process are discussed, thus highlighting those, which are amenable to formal modelling as well as those where theoretical approaches are still elusive. This discussion is then continued in the second article by Michael Zhang [2], who presents experimental and computational approaches to understand gene regulatory networks. The understanding of these networks is one of the most difficult problems, especially for eukaryotes, which feature complex promoters. The identification of regulatory elements and regulatory motifs in such promoters is then discussed in a general probabilistic framework by Erik van Nimwegen [3]. In his article Erik elucidates that many seemingly different bioinformatics methods used today are just different facets of a single integrated probabilistic theory.

A review of computational and statistical methods to reconstruct cellular networks is given by Florian Markowetz and Rainer Spang [4]. The review deals with conditional independence models including Gaussian graphical models and Bayesian networks and discusses probabilistic and graph-based methods for data from experimental interventions. This is the one article that is not based on a presentation given at the Summer School, but instead was included because it really had been lacking there. Harmen Bussemaker et al. [5] then discusses two methods to analyse transcriptional responses in mRNA expression levels. An analysis on the pathway-level of differential expression based on prior information can be considerably more sensitive to subtle changes in gene expression than gene-level analysis. The statistics of gene regulation is further elucidated by Michael Lassig [6]. In his review he covers the biophysical, bioinformatics, and evolutionary aspects of gene regulation.

Graph theoretical concepts and relationship to biological systems are further discussed by Wolfgang Huber et al [7]. This review gives a brief introduction into some of the concepts and their areas of application in molecular biology. Subsequently, Thomas Schlitt and Alvis Brazma [8] describe some approaches to gene regulatory network modelling. Examples for network parts lists, network topology models, network control logic models, and dynamic models are given, as well as a discussion of simple network dynamics.

It is our hope that this volume will serve as an introduction for researchers moving into the field of regulatory genomics and, in particular, will provide interested students with a resource, where to find the necessary background material to put current developments in a larger scientific context.
